# Health-related quality of life in adults with epilepsy: the effect of age, age at onset and duration of epilepsy in a multicentre Italian study

**DOI:** 10.1186/1471-2377-11-33

**Published:** 2011-03-10

**Authors:** Valeria Edefonti, Francesca Bravi, Katherine Turner, Ettore Beghi, Maria Paola Canevini, Monica Ferraroni, Ada Piazzini

**Affiliations:** 1 Sezione di Statistica Medica e Biometria 'Giulio A. Maccacaro', Dipartimento di Medicina del Lavoro 'Clinica del Lavoro L. Devoto', Università degli Studi di Milano, Milan, Italy; 2 Dipartimento di Epidemiologia, Istituto di Ricerche Farmacologiche 'Mario Negri', Milan, Italy; 3 UO Neurologia 2, Centro Epilessia, Azienda Ospedaliera San Paolo, Milan, Italy; 4 Dipartimento di Neuroscienze, Istituto di Ricerche Farmacologiche 'Mario Negri', Milan, Italy; 5 Dipartimento di Medicina, Chirurgia e Odontoiatria, Università degli Studi di Milano, Milan, Italy

## Abstract

**Background:**

The potential effect of age-related factors on health-related quality of life (HRQOL) of patients with epilepsy has rarely been analyzed in the literature.

**Methods:**

We examined this association in a selected population of 815 adults with epilepsy recruited in the context of a multicentre study for the evaluation of Epi-QoL, one of the first Italian epilepsy-specific measures of HRQOL for adults with epilepsy. The Epi-QoL is a 46-item self-administered questionnaire focusing on six domains, which was successfully tested for reproducibility and validity. Ordinary least-squares regression models were used to assess the relationships between age-related factors (patient's age, age at seizure onset, and duration of epilepsy) and overall Epi-QoL score, controlling for the effect of potential confounders. We fitted simple regression models including each age-related factor alone to assess the independent role of each factor on the overall Epi-QoL score. We also fitted multiple regression models including pairs of age-related factors solely, as well as one or two age-related factors together with the same set of confounders.

**Results:**

Simple regression models showed that age and duration of epilepsy were significant negative predictors of the overall Epi-QoL score: the higher was each age-related factor, the lower was the overall Epi-QoL score; age at onset alone was a nonsignificant predictor of the overall Epi-QoL score. Multiple regression models including two age-related factors solely showed that duration of epilepsy was still a significant negative predictor of the overall Epi-QoL score in both pairwise models, whereas age was a significant negative predictor only in the model including age at onset. Age at onset emerged as a significant positive predictor of the overall Epi-QoL score only in the model including age: the higher was age at onset, the higher was the overall Epi-QoL score. Adjusted regression models including either one or two age-related factors and controlling for the selected confounding variables showed that the age-related factors had no significant effect on the overall Epi-QoL score anymore.

**Conclusions:**

If no other known correlates of the overall Epi-QoL score are considered, age and duration of epilepsy can be expected to have a significant negative association with HRQOL in epilepsy (with the effect of duration being stronger and more consistent across models than the one of age), whereas age at onset is a positive predictor of the overall HRQOL of limited significance. However, demographic and clinical factors, such as seizure frequency in the preceding 12 months, may provide a better explanation of HRQOL in epilepsy.

## Background

Patients with epilepsy are at increased risk for poor health-related quality of life (HRQOL), given the impact of this disorder on psycho-social dimensions that contribute to form the overall perception of an individual's position in life [1-3].

During the past two decades considerable research has been devoted to developing measures of HRQOL for epilepsy patients [4,5], and to examining the impact of demographic (e.g. age, sex, education, socio-economic status), clinical (e.g. seizure frequency, seizure type, duration of epilepsy, age at onset, type of drugs and number), comorbid psychiatric (e.g. anxiety, depression), and psycho-social (e.g. stigma, social support, self-efficacy) factors on quality of life [3,6-8].

The potential effect of age-related factors, including age, age at onset of seizures and duration, on HRQOL of patients with epilepsy is a source of conceptual and methodological concern. Potential interest lies in the evaluation of the independent contribution of each of these factors on HRQOL, as well as of their joint effect. Including two age-related factors in the same model allows to estimate the effect of interest adjusting for the effect of the remaning age-related factor, and this may provide results that are different from the ones of simple models including only the age-related factor of interest, in consideration of confounding phenomena. For instance, in adult patients, since several epilepsies start early in life, a longer duration of the disorder is heavily confounded by ageing, which itself is associated with cognitive decline and poor HRQOL [9]. However, the linear relationship that links the three age-related factors (say, epilepsy duration = age - age at onset) prevents the inclusion in the same model of all the factors as continuous variables. The major implication of this issue is that one can get a good overall picture on age-related morbidity and HRQOL only from the joint evaluation of the three models including each available pair of age-related factors.

The relationship between age-related factors and HRQOL in epilepsy patients has rarely been analyzed in the literature. We identified only one study [10] devoted to assessing the effects of age, age at onset and epilepsy duration on HRQOL. Some results on age-related morbidity were obtained as a by-product from studies intended to address other research questions on HRQOL, measured according to a variety of validated instruments, in various subpopulations [11-30]. Moreover, few research efforts have been devoted to understanding the cognitive burdens associated with increasing age [9,31], or duration of epilepsy [31-33], and the comorbid interictal psychiatric symptoms associated with duration of epilepsy too [28].

In the present paper, we examine the independent and joint effects of age, age at onset and duration of epilepsy on HRQOL in adult patients with this disorder recruited in a multicentre Italian study based on one of the first validated epilepsy-specific questionnaires developed in Italy [34]. Although these factors are unmodifiable, they may play a crucial role in the identification of epilepsy patients at higher risk of poor HRQOL and of targeted clinical, psychological and social care.

## Methods

### Subjects

The present analysis was based on data from a multicentre study including 815 subjects and evaluating the Epi-QoL, one of the first epilepsy-specific instruments developed in Italy to measure HRQOL in clinical practice. Details were given elsewere [34]. Briefly, the 24 secondary and tertiary Italian centers for the care of epilepsy participating in the study were asked to recruit up to 30 consecutive patients meeting the following inclusion criteria: age 18 or older; diagnosis of epilepsy (at least two unprovoked seizures 24 hours apart) [35]; presence of idiopathic, cryptogenic, or symptomatic epilepsy according to the ILAE syndromic classification [36]; good compliance with treatment and study participation; informed consent; and ability to read and understand the study questions. A semi-structured questionnaire including information on socio-demographic characteristics and clinical variables was administered to outpatients by trained interviewers. The study was approved by the local ethics committees. HRQOL was evaluated through the Epi-QoL instrument.

### Measures

The Epi-QoL is a 46-item self-administered questionnaire focusing on 6 domains identified by an expert panel: Physical Functioning, Cognitive Functioning, Emotional Well-Being, Social Functioning, Seizure Worry, and Medication Effects. Subjects have to rate their HRQOL on a 6-point Likert scale format: 'almost always' (1), 'very frequently' (2), 'sometimes' (3), 'a bit' (4), 'very little' (5), 'not at all' (6). The instrument was successfully tested for reproducibility and validity and showed fairly good psychometric properties [34].

### Statistical analysis

#### Variables of interest

In accordance with the defined aim of the analysis, we carried out a preliminary identification of a set of variables of interest. This set includes the overall Epi-QoL score as a measure of HRQOL, the three mentioned age-related factors (age, age at onset of epilepsy, and duration of epilepsy) as main variables of interest, and a set of other factors identified by our panel of experts as potential confounding variables. The overall Epi-QoL score was calculated for each patient as the sum of the scores attributed to each questionnaire's item, after reverse coding was applied to those scores corresponding to questions that were positively formulated. The higher the score, the greater the HRQOL perceived [34]. Age, age at onset and duration were measured in years. Age and age at onset were obtained from the initial clinical interview and previous records. Duration of epilepsy was computed by subtracting age at onset from age. The set of potential confounders includes the following variables: geographic area of the study center, gender, education, seizure frequency in the preceding 12 months, etiology, number of medications, intellectual functions, and psychiatric disturbances. Details on the distribution of the confounders in the study sample were given in the Results section and elsewhere [34].

#### Descriptive analyses

We computed a kernel density estimate of the distribution of each age-related factor and of the overall Epi-QoL score to improve histogram-like representations of the original data. We adopted a Gaussian smoothing kernel scaled such that the smoothing bandwidth is the standard deviation of the kernel [37]. We calculated the Spearman rank correlation coefficient between each pair of variables included in the identified set of interest. Given the large number of subjects (N = 815), values greater or equal to 0.069 in absolute value were retained as statistically significant at the *α *= 005 level [38].

#### Regression models

Ordinary least-squares regression models were used to assess the relationships between age-related factors, potential confounders and overall Epi-QoL score, with overall Epi-QoL score being the dependent variable, and age-related factors and potential confounders the independent variables. For each age-related factor, we ran a simple regression model, followed by a multiple model including all the potential confounders. We also fitted models including pairs of age-related variables solely and in combination with all the potential confounders.

To improve interpretability of the regression coefficients, we considered the age-related factors within each of the previous models as continuous (in years) as well as categorical variables, with categories defined as in the provided tables.

Checks of regression diagnostics were carried out for all the fitted models and revealed only limited adherence to the model assumption on normality of the error. However, as the p-values obtained from the corresponding models with a Box-Cox transformation of the response variable (*y*^*λ *= 2.3^) [39,40] were similar to those obtained from the original models, we decided to present the results obtained from the original models with no transformation of the dependent variable.

Calculations were performed using the open-source statistical computing environment R [41,42], and its libraries "MASS" [43] and "faraway" [44].

## Results

The demographic and clinical characteristics of the study sample are described elsewhere [34]. Briefly, patients had a median age of 36 years, a median epilepsy duration of 16 years, and a median age at onset of seizures of 16 years. Almost 40% of patients had no seizures in the preceding 12 months, 25.5% had from 1 to 5 seizures, 13% from 6 to 20, and 21.7% had more than 20 seizures. Most of the patients had focal (74.3%) or generalized (22.2%) epilepsy, were in remission for more than a year (53.4%) or had non-drug-resistant seizures (20.3%), and were in monotherapy (54.1%) or polytherapy (44.3%).

**Figure 1:**
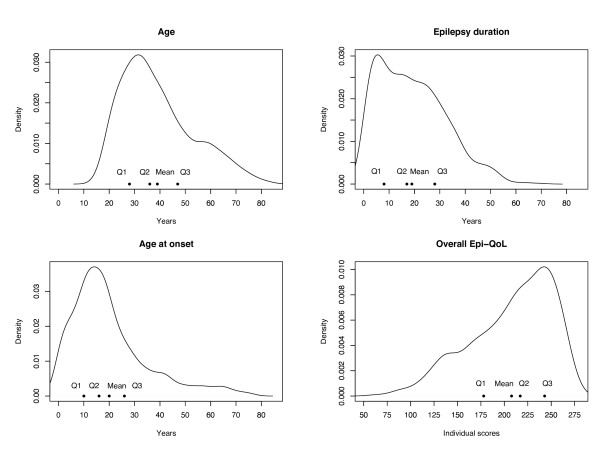
**Kernel density estimation plot of the univariate distributions of the age-related factors and overall Epi-QoL score**. Density estimates were plotted along with quartiles and means of each variable. In each plot, Q1, Q2 and Q3 indicated the first, second (median), and third quartile, respectively. The bandwidths were about 3.34, 3.11, 2.81, 10.28 for age, duration, age at onset, and overall Epi-QoL score, respectively.

Figure 1 shows the kernel density estimation plot of the univariate distributions of age-related factors and overall Epi-QoL score, plotted along with the quartiles and the mean of each variable. The density estimates provide an indication of the general tendency of each variable to distribute on the range of its possible values. Here, all the density estimates emerged to be highly skewed, with the age-related densities that were skewed to the right and the overall Epi-QoL score density that was skewed to the left. Accordingly, in the age-related factor plots, the means were larger than the corresponding medians (Q2), whereas in the overall Epi-QoL score plot the mean was smaller than the corresponding median (Q2).

Table 1 shows the Spearman rank correlation coefficients, *ρ*, between the identified variables of interest. As expected, the correlations among the age-related variables were high in absolute value, with the smallest absolute value given by 0.39 and the highest given by 0.59. Age was positively correlated with age at onset (*ρ *= 0.42) and duration of epilepsy (*ρ *= 0.39), the latter two variables were negatively correlated (*ρ *= -0.59). The overall Epi-QoL score was correlated with the following potential confounders: seizure frequency in the preceding 12 months, psychiatric disturbances, number of drugs, and intellectual functions, but it was correlated only weakly with the age-related factors (see [34] and the beginning of this section for details on these variables).

**Table 1:** Spearman rank correlation coefficients between age-related variables, overall Epi-QoL score and demographic and clinical characteristics (N = 815).^a^

	A	Ao	D	O-Epi	Sf	Pd	G	Nd	If	Ed	Ga	Et
A	1.00											

Ao	**0.42**	1.00										

D	**0.39**	**-0.59**	1.00									

O-Epi	**-0.08**	0.05	**-0.12**	1.00								

Sf	**0.08**	-0.06	**0.11**	**-0.41**	1.00							

Pd	**0.11**	0.02	**0.07**	**-0.21**	**0.08**	1.00						

G	0.06	**0.15**	**-0.12**	**0.13**	-0.06	-0.06	1.00					

Nd	**0.09**	**-0.23**	**0.31**	**-0.29**	**0.40**	**0.08**	-0.03	1.00				

If	-0.05	**0.19**	**-0.20**	**0.21**	**-0.11**	**-0.14**	**-0.01**	**-0.18**	1.00			

Ed	**0.09**	0.04	0.05	-0.04	0.04	0.04	0.05	**0.08**	-0.04	1.00		

Ga	**-0.08**	**-0.09**	0.03	**-0.13**	**0.09**	-0.02	0.03	0.01	-0.01	-0.00	1.00	

Et	**0.08**	-0.04	**0.07**	**-0.08**	**0.07**	-0.05	0.03	0.07	**-0.14**	0.03	-0.05	1.00

^a^Values greater or equal to 0.069 in absolute value were retained as statistically significant at the *α *= 0.05 level and were shown in bold typeface.

A: Age; Ao: Age at onset; D: Duration of epilepsy; O-Epi: Overall Epi-QoL score; Sf: Seizure frequency (12 months); Pd: Psychiatric disturbances; G: Gender; Nd: Number of drugs; If: Intellectual functions; Ed: Education; Ga: Geographic area; Et: Etiology.

Results from the fitted regression models with continuous age-related factors are summarized below. Simple regression models including just one age-related factor showed that age at onset was not a significant predictor (*β *= 0.13, p = 0.188), whereas age and duration of epilepsy were significant negative predictors of the overall Epi-QoL score (*β *= -0.24, p = 0.025, and *β *= -0.46, p < 0.001, respectively). Multiple regression models including two age-related factors showed that duration of epilepsy was still a significant negative predictor of the overall Epi-QoL score in both pairwise models (with age: *β *= -0.42, p < 0.001, with age at onset: *β *= -0.51, p < 0.001), and its effect was comparable in size to the one that emerged from the simple model including duration only. Age was a significant predictor of the overall Epi-QoL score only in the model including age at onset (*β *= -0.51, p < 0.001), whereas it was nonsignificant when duration was included in the model (*β *= -0.10, p = 0.406). In both models, the coefficient beta for age was still negative, and it was stronger than in the simple age-model, when age at onset was included in the model. Age at onset emerged as a significant predictor of the overall Epi-QoL score in the model including age (*β *= 0.42, p < 0.001), but not when duration was included (*β *= -0.10, p = 0.406). When significant, age at onset had a positive effect on the overall Epi-QoL score. Adjusted regression models including either one or two age-related factors, as well as the selected confounding variables, showed that the age-related factors had no significant effect on the overall Epi-QoL score.

Table 2 and 3 summarize the results coming from the fitted regression models with categorical age-related factors. Type-1 models include only the age-related variables (either one or two of them) as predictors of the overall Epi-QoL score. Type-2 models include the age-related variables (either one or two of them) and the selected potential confounders (as listed in the left outer column of the tables). Results were generally consistent with those obtained from the regression models including the continuous age-related factors, but the magnitude of the effects was more evident. Categories 40- < 50 and 50- < 60 years for age, 30- < 40 and ≥ 40 years for duration of epilepy, and 10- < 25 and ≥ 55 for age at onset emerged as statistically significant across the fitted models. In detail, type-1 simple models showed that the decrease in the overall Epi-QoL score given, say, by a chronological age between 50 and 60 years (compared to being less than 30 years) was of about 17 points (*β *= -16.63, p = 0.002), and that the decrease given by a duration of epilepsy of, say, more than 40 years (compared to a duration of less than 10 years) was of about 19 points (*β *= 19.37, p = 0.002). Moreover, an age at onset between 10 and 25 years (compared to having an onset at 10 years or less) provided a significant increase in the overall Epi-QoL score of about 8 points (*β *= 7.78, p = 0.043). In the type-1 multiple models, duration of epilepsy remained significant and equally strong, age at onset attained some significance, whereas the association with age either remained stable and significant or became weaker and nonsignificant. Type-2 models showed that none of the age-related variables had significant effects on the overall Epi-QoL score, after adjusting for the selected confounders. For both continuous and categorical age-related models, seizure frequency in the preceding 12 months, psychiatric disturbances, number of medications, geographic area of the study center, gender, intellectual functions, and education explained most of the overall Epi-QoL score, and were, therefore, more relevant determinants of HRQOL (measured according to the overall Epi-QoL score) than age-related factors. This was evident in the comparison between the adjusted R^2^s (percentage of total variability in the outcome that is accounted for by the entire set of predictors) across type-1 and corresponding type-2 models: type-2 models provided approximately 25% of the explanation for the overall Epi-QoL score, compared with 0.4, 1, and 2% for models with age at onset, age, and duration of epilepsy alone, respectively. Most of this 25% was attributable to the seizure frequency in the preceding 12 months (data not shown).

**Table 2:** Ordinary least-squares regression models with categorical age-related factors as main effects: results from models including single age-related factors (N = 815).^a^

Age-related factor	N	%			N	%
Age				Age at onset		

<30	238	29.2		<10	188	23.1

30-<40	246	30.2		10-<25	407	49.9

40-<50	149	18.3		25-<55	180	22.1

50-<60	93	11.4		≥ 55	40	4.9

≥60	89	10.9				

Duration of epilepsy						

<10	244	29.9				

10-<20	207	25.4				

20-<30	184	22.6				

30-<40	122	15.0				

≥ 40	58	7.1				

						

Predictor variable	Type-1 Model			Type-2 Model		

	*β*	SE	p	*β*	SE	p

**Age**						

**30-<40**	**-4.68**	**3.95**	**0.237**	**0.39**	**3.51**	**0.911**

**40-<50**	**-10.52**	**4.54**	**0.021**	**-4.13**	**4.13**	**0.318**

**50-<60**	**-16.63**	**5.31**	**0.002**	**-8.94**	**4.83**	**0.065**

≥ **60**	**-5.44**	**5.40**	**0.314**	**1.37**	**5.08**	**0.787**

Seizure frequency in preceding 12 months						

1-5 vs seizure free				-18.59	3.48	<0.001

6-20 vs seizure free				-23.65	4.47	<0.001

≥20 vs seizure free				-33.12	4.02	<0.001

Psychiatric disturbances						

yes vs no				-19.97	4.03	<0.001

Gender						

male vs female				8.22	2.71	0.002

Therapy						

monotherapy vs polytherapy				9.24	3.03	0.002

no therapy vs polytherapy				17.86	10.91	0.102

Intellectual functions						

compromised vs normal				-12.35	4.10	0.003

Education						

9-13 years vs <9 years				6.95	3.14	0.027

≥ 14 years vs <9 years				9.97	4.17	0.017

Geographic area						

central Italy vs northern Italy				-2.47	3.29	0.452

southern Italy vs northern Italy				-12.93	3.45	<0.001

Etiology						

cryptogenic vs symptomatic				4.09	3.08	0.184

idiopathic vs symptomatic				3.11	3.85	0.419

						

Intercept	213.51	2.82	<0.001	216.65	5.49	<0.001

*R*^2^	0.01			0.25		

						

**Age at onset**						

**10-<25**	**7.78**	**3.84**	**0.043**	**-2.04**	**3.45**	**0.555**

**25-<55**	**3.17**	**4.55**	**0.486**	**-6.93**	**4.18**	**0.098**

≥ **55**	**13.54**	**7.59**	**0.075**	**5.67**	**6.90**	**0.412**

Seizure frequency in preceding 12 months						

1-5 vs seizure free				-18.86	3.50	<0.001

6-20 vs seizure free				-23.43	4.48	<0.001

≥ 20 vs seizure free				-33.22	4.02	<0.001

Psychiatric disturbances						

yes vs no				-20.20	4.01	<0.001

Gender						

male vs female				8.040	2.720	0.003

Therapy						

monotherapy vs polytherapy				10.24	3.07	<0.001

no therapy vs polytherapy				18.66	10.93	0.088

Intellectual functions						

compromised vs normal				-12.70	4.15	0.002

Education						

9-13 years vs <9 years				7.86	3.06	0.010

≥ 14 years vs <9 years				11.73	4.08	0.004

Geographic area						

central Italy vs northern Italy				-2.14	3.28	0.514

southern Italy vs northern Italy				-12.25	3.38	<0.001

Etiology						

cryptogenic vs symptomatic				4.71	3.10	0.129

idiopathic vs symptomatic				3.05	3.88	0.431

						

Intercept	202.43	3.18	<0.001	215.86	5.04	<0.001

*R*^2^	0.01			0.25		

						

**Duration of epilepsy**						

**10-<20**	**2.32**	**4.09**	**0.571**	**4.42**	**3.65**	**0.226**

**20-<30**	**-3.04**	**4.22**	**0.471**	**4.25**	**3.88**	**0.273**

**30-<40**	**-13.53**	**4.80**	**0.005**	**2.13**	**4.48**	**0.634**

≥ **40**	**-19.37**	**6.32**	**0.002**	**-6.78**	**5.84**	**0.246**

Seizure frequency in preceding 12 months						

1-5 vs seizure free				-18.15	3.49	<0.001

6-20 vs seizure free				-23.06	4.48	<0.001

≥ 20 vs seizure free				-32.12	4.03	<0.001

Psychiatric disturbances						

yes vs no				-20.18	4.02	<0.001

Gender						

male vs female				8.48	2.71	0.002

Therapy						

monotherapy vs polytherapy				9.88	3.15	0.002

no therapy vs polytherapy				20.22	10.95	0.065

Intellectual functions						

compromised vs normal				-12.13	4.12	0.003

Education						

9-13 years vs <9 years				6.89	3.07	0.025

≥ 14 years vs <9 years				10.16	4.09	0.013

Geographic area						

central Italy vs northern Italy				-2.45	3.30	0.459

southern Italy vs northern Italy				-13.32	3.43	<0.001

Etiology						

cryptogenic vs symptomatic				4.54	3.08	0.141

idiopathic vs symptomatic				3.91	3.78	0.301

						

Intercept	211.18	2.77	<0.001	212.07	5.62	<0.001

*R*^2^	0.02			0.25		

^a^Type-1 models include only the age-related variables as predictors of the overall Epi-QoL score. Type-2 models include the age-related variables and the selected potential confounders (as listed in the left outer column of the table). All variables included in the models were shown.*β*: unstandardized regression coefficient; SE: standard error.

**Table 3:** Ordinary least-squares regression models with categorical age-related factors as main effects: results from models including available pairs of age-related factors (N = 815).^a^

Predictor variable	Type-1 Model			Type-2 Model		
	*β*	SE	p	*β*	SE	p

**Age**						

**30-<40**	**-4.82**	**4.00**	**0.228**	**1.03**	**3.57**	**0.773**

**40-<50**	**-10.98**	**4.75**	**0.021**	**-2.79**	**4.33**	**0.520**

**50-<60**	**-18.41**	**5.57**	**<0.001**	**-7.85**	**5.10**	**0.124**

**≥ 60**	**-12.32**	**6.61**	**0.063**	**0.08**	**6.13**	**0.989**

**Age at onset**						

**10-<25**	**7.01**	**3.83**	**0.068**	**-2.03**	**3.45**	**0.557**

**25-<55**	**7.88**	**4.75**	**0.097**	**-5.51**	**4.44**	**0.215**

**≥ 55**	**20.89**	**9.05**	**0.021**	**5.80**	**8.19**	**0.479**

Seizure frequency in preceding 12 months						

1-5 vs seizure free				-18.77	3.50	<0.001

6-20 vs seizure free				-23.76	4.49	<0.001

≥ 20 vs seizure free				-33.54	4.03	<0.001

Psychiatric disturbances						

yes vs no				-19.77	4.03	<0.001

Gender						

male vs female				8.13	2.73	0.003

Therapy						

monotherapy vs polytherapy				9.61	3.10	0.002

no therapy vs polytherapy				17.06	10.97	0.120

Intellectual functions						

compromised vs normal				-12.84	4.15	0.002

Education						

9-13 years vs <9 years				7.33	3.15	0.020

≥ 14 years vs <9 years				10.84	4.20	0.010

Geographic area						

central Italy vs northern Italy				-2.32	3.29	0.480

southern Italy vs northern Italy				-12.71	3.46	<0.001

Etiology						

cryptogenic vs symptomatic				4.58	3.11	0.141

idiopathic vs symptomatic				2.72	3.90	0.486

						

Intercept	208.32	3.95	<0.001	217.69	5.87	<0.001

*R*^2^	0.01			0.25		

						

**Age**						

**30-<40**	**-2.40**	**4.12**	**0.561**	**-0.77**	**3.67**	**0.834**

**40-<50**	**-5.11**	**4.89**	**0.296**	**-4.50**	**4.44**	**0.311**

**50-<60**	**-8.80**	**5.81**	**0.130**	**-8.00**	**5.28**	**0.130**

**≥ 60**	**0.99**	**5.75**	**0.863**	**3.49**	**5.40**	**0.519**

**Duration of epilepsy**						

**10-<20**	**2.54**	**4.12**	**0.538**	**4.62**	**3.66**	**0.207**

**20-<30**	**-1.91**	**4.37**	**0.663**	**5.18**	**4.01**	**0.197**

**30-<40**	**-10.57**	**5.17**	**0.041**	**4.83**	**4.79**	**0.314**

**≥ 40**	**-17.02**	**6.89**	**0.014**	**-5.02**	**6.34**	**0.429**

Seizure frequency in preceding 12 months						

1-5 vs seizure free				-18.19	3.50	<0.001

6-20 vs seizure free				-23.33	4.49	<0.001

≥ 20 vs seizure free				-32.64	4.04	<0.001

Psychiatric disturbances						

yes vs no				-20.07	4.04	<0.001

Gender						

male vs female				8.49	2.72	0.002

Therapy						

monotherapy vs polytherapy				9.74	3.15	0.002

no therapy vs polytherapy				18.63	10.97	0.090

Intellectual functions						

compromised vs normal				-12.44	4.15	0.003

Education						

9-13 years vs <9 years				7.15	3.15	0.024

≥ 14 years vs <9 years				10.28	4.17	0.014

Geographic area						

central Italy vs northern Italy				-2.51	3.31	0.449

southern Italy vs northern Italy				-13.21	3.47	<0.001

Etiology						

cryptogenic vs symptomatic				4.49	3.09	0.146

idiopathic vs symptomatic				3.30	3.89	0.397

						

Intercept	212.82	3.43	<0.001	213.15	6.09	<0.001

*R*^2^	0.02			0.25		

						

**Age at onset**						

**10-<25**	**1.08**	**4.22**	**0.797**	**-3.59**	**3.75**	**0.339**

**25-<55**	**-5.21**	**5.23**	**0.319**	**-8.17**	**4.76**	**0.086**

**≥ 55**	**3.64**	**8.49**	**0.668**	**5.29**	**7.78**	**0.497**

**Duration of epilepsy**						

**10-<20**	**1.58**	**4.34**	**0.715**	**4.32**	**3.86**	**0.263**

**20-<30**	**-3.86**	**4.69**	**0.411**	**3.45**	**4.27**	**0.418**

**30-<40**	**-14.47**	**5.46**	**0.008**	**0.54**	**5.07**	**0.914**

**≥ 40**	**-20.60**	**7.02**	**0.003**	**-9.04**	**6.48**	**0.164**

Seizure frequency in preceding 12 months						

1-5 vs seizure free				-18.49	3.50	<0.001

6-20 vs seizure free				-23.19	4.48	<0.001

≥ 20 vs seizure free				-32.71	4.03	<0.001

Psychiatric disturbances						

yes vs no				-19.75	4.03	<0.001

Gender						

male vs female				8.34	2.72	0.002

Therapy						

monotherapy vs polytherapy				9.99	3.14	0.002

no therapy vs polytherapy				18.37	10.97	0.094

Intellectual functions						

compromised vs normal				-12.74	4.15	0.002

Education						

9-13 years vs <9 years				7.26	3.11	0.020

≥ 14 years vs <9 years				11.06	4.15	0.008

Geographic area						

central Italy vs northern Italy				-2.46	3.30	0.457

southern Italy vs northern Italy				-13.32	3.46	<0.001

Etiology						

cryptogenic vs symptomatic				5.231	3.106	0.093

idiopathic vs symptomatic				3.264	3.888	0.401

						

Intercept	212.22	5.21	<0.001	215.85	7.03	<0.001

*R*^2^	0.03			0.26		

^a^Type-1 models include only the age-related variables as predictors of the overall Epi-QoL score. Type-2 models include the age-related variables and the selected potential confounders (as listed in the left outer column of the table). All variables included in the models were shown.*β*: unstandardized regression coefficient; SE: standard error.

## Discussion

The present analysis is devoted to the elucidation of the role of age, age at seizure onset, and epilepsy duration on HRQOL in an Italian multicentre study on epilepsy patients based on the validated and specific Epi-QoL questionnaire. It emerges that, if no other known correlates of the overall Epi-QoL score are considered, age and duration of epilepsy can be expected to have a significant consistent negative association, and age at onset a positive association of limited significance (i.e. significant in one model only), with HRQOL in epilepsy. However, none of the age-related variables showed a significant effect on the overall Epi-QoL score, after adjusting for a selected set of confounders including demographic and clinical factors, and, in particular, the seizure frequency in the preceding 12 months.

The main conclusion of a modest role of age-related factors as determinants of HRQOL is consistent with the vast majority of the literature reports on the determinants of HRQOL in epilepsy patients [11-30], including the only available study published on the same topic to our knowledge [10], where the effects of age at onset and duration were no longer significant, after adding Profile of Mood States Depression/Dejection, Adverse Events Profile, comorbidities, and antiepileptic drugs to the regression models.

Nonsignificance of age-related factors has rarely been reported in simple models [10,12], but is more common when results come from multiple models including age-related factors and other clinical variables, such as measures of psychiatric comorbidity, number of antiepileptic medications, and seizure severity, as in Szaflarski and coauthors [10,11,15,16,26]. The instrument used to measure HRQOL may also play a role, as specific measures were found to be more sensitive in detecting variation in age at onset and other clinical variables than generic measures [14].

Significant but modest effects of age-related factors on HRQOL in epilepsy patients have been reported in other papers, even after adjustment for other known important determinants [14,17-22,24,25]. However, some inconsistencies emerged in the sign of the effect of the age-related factors on HRQOL.

In our analysis, age was a relevant determinant of HRQOL: its negative impact on HRQOL emerged from all the three type-1 models, with statistical significance of the coefficients given in two of them. This is in accordance with findings from an analysis based on adolescents [19], where older patients (14-17 years), independent of epilepsy severity, reported worse overall HRQOL than did their younger counterparts, and with several studies on chronic health problems and HRQOL [45]. However, in one of the few reported studies that focuses on age in epilepsy adults, Pugh and colleagues [18] reported that older adults (65 years and older) seemed to have less compromised QOL (as measured using a generic health status measure, the SF-36) than young (18-40 years) and middle-aged (41-64 years) adults. Ageing is associated with a mean decline in learning and memory performances, and, although this decline was apparently similar for epilepsy patients and healthy subjects, the former group reached poor performance levels much earlier than the latter, as epilepsy patients may fail to build up adequate learning and memory performance during childhood and adolescence [9]. This may have important implications in terms of a limited HRQOL. Moreover, age is generally associated with an increased number of chronic health problems, and multiple chronic health problems are significantly associated with a reduced HRQOL, in particular with reduced levels of physical and emotional functioning [45]. In patients with a previous diagnosis of epilepsy, the ability of coping with new chronic health problems and their consequences, together with epilepsy severity, may be crucial in the evaluation of the role of age on HRQOL.

From our analysis, it emerged that duration of epilepsy had a consistent significant negative effect on HRQOL in both single and pairwise unadjusted models. This is still in agreement with findings from studies on epilepsy adolescents, where a longer duration of epilepsy had a significant negative impact on the memory and concentration subscale of QOLIE-AD-48 [19] and on the overall HRQOL [17], and with various reports on the effects of disorder duration for other chronic conditions, such as Parkinson's disease [46]. However, in Szaflarski and coauthors [10], duration had a significant positive effect on HRQOL in the simple regression model. Even in presence of working adjustments of the social and psychological consequences of the disorder and coping mechanisms, cognitive decline and emotional-behavioral distress, together with a potentially increased number of drugs, may lead epilepsy subjects to give a poorer evaluation of HRQOL. Indeed, some studies assessing verbal learning and memory skill of epilepsy patients with ad-hoc inventories suggested a slow cognitive decline with increased duration of epilepsy [32,33]. A relevant investigation [28] demonstrated that increasing years of duration of temporal lobe epilepsy was modestly associated with increased and generalized self-reported emotional-behavioral distress, even after adjustment for potentially confounding clinical variables (including age of onset), and that this comorbid emotional-behavioral distress was significantly associated with a poorer HRQOL.

Finally, in our paper, age at onset emerged as a positive predictor of limited significance (in one model out of three) for the overall Epi-QoL. Accordingly, consistent evidence has been reported from studies on epilepsy children and adolescents, with two studies [14,17] suggesting that an earlier age of epilepsy onset was associated with poorer HRQOL. In one study on adults who had operations for refractory extratemporal epilepsy [24], onset at an older age predicted a minimal increase in the overall health scores, but not in the total HRQOL score, in both single and multiple models. On the contrary, the paper by Szaflarski and coauthors [10] and two others [21,22] suggested that adding years to age at onset decreased HRQOL. In detail, in the same population of young epilepsy adults with mild intellectual disabilities, epilepsy/disability onset in adolescence (after the age of 10 years, compared with disability onset during childhood) was associated with poorer HRQOL, both independently and in interaction with neuroticism [21,22]. The first set of studies, including our analysis, provided data supporting the association between poorer seizure control, diminished neuropsychological functioning, and low HRQOL in patients with an earlier onset of epilepsy. However, the role of age at epilepsy onset deserves further attention, especially in its joint effect with age, in consideration of how epilepsy can interfere with brain maturation and then can affect cognitive functioning in the long term [9].

A possible explanation of the differences in significance and sign of the age-related beta coefficients across the papers may be found in the different set of covariates included as potential confounders in the regression models, and in the different populations under consideration. For instance, while Szaflarski and coauthors [10] restricted the analyses to adult patients with medication-resistant epilepsy from a US epilepsy monitoring center, we based our analyses on the overall study population of adult patients recruited in secondary and tertiary Italian centers for the care of epilepsy. When we selected the subgroup of medication-resistant epilepsy patients and carried out the same pool of analyses, the significant associations previously identified in the type-1 models disappeared, and none of the age-related factors were found to be relevant in the explanation of HRQOL. Although nonsignificant, the signs of the linear associations between age-related factors and HRQOL were more similar to those in Szaflarski et al. [10]: age at onset emerged as negatively associated with HRQOL across all the fitted models, and the estimated beta coefficient for duration changed its sign to positive in the pairwise model with age (data not shown).

## Conclusions

The Epi-QoL questionnaire was developed with the scope of creating a specific instrument to address needs and expectations of the Italian epilepsy adults in their evaluation of HRQOL. It was validated successfully through a multicentre study, showing fairly good psychometric properties. This paper attempts to use the promising results from this study to address a specific research question on the role of age-related factors as determinants of HRQOL, as measured by the overall Epi-QoL score. The main conclusion is that age, age at onset, and duration of epilepsy have only a limited role in determining the overall HRQOL: age and duration of epilepsy show a generally significant negative effect (with the effect of duration being stronger and more consistent across models than the one of age), and age at onset a positive effect of limited significance, when considered alone or with another age-related factor; however, when other correlates of the overall Epi-QoL score are included in the regression models, any significant effect of the age-related factors disappears.

## Competing interests

The authors declare that they have no competing interests.

## Authors' contributions

VE conceived and carried out the statistical analysis, and drafted the manuscript. FB carried out the preliminary checks on the quality of the data, helped in the review of the literature, and participated in the statistical analysis. KT participated in the study design and coordination. EB conceived the study, and participated in its design. MPC conceived the study, and participated in its design. MF identified the problem of interest, supervised and supported the statistical analysis. AP conceived the study, participated in its design and coordination, and helped to draft the manuscript. All authors read and approved the final manuscript.

## Pre-publication history

The pre-publication history for this paper can be accessed here:

http://www.biomedcentral.com/1471-2377/11/33/prepub
